# Non invasive assessment of liver fibrosis in chronic hemodialysis patients with viral hepatitis C

**DOI:** 10.11604/pamj.2015.22.273.2311

**Published:** 2015-11-23

**Authors:** Mohamed Arrayhani, Tarik Sqalli, Nada Tazi, Randa El Youbi, Safae Chaouch, Nourdin Aqodad, Sidi Adil Ibrahimi

**Affiliations:** 1 Nephrology Department, University Hospital Hassan II, Fez, Morocco; 2 Faculty of Medicine and Pharmacy, Fez, Morocco; 3 Gastroenterology Department, University Hospital Hassan II, Fez, Morocco

**Keywords:** APRI, fib4, fibroscan, forns, hepatitis C virus, liver fibrosis, chronic hemodialysis

## Abstract

The liver biopsy has long been the "gold standard" for assessing liver fibrosis in patients with hepatitis C. It's an invasive procedure which is associated with an elevated bleeding, especially in chronic hemodialysis patients. Main goal is to assess liver fibrosis in chronic hemodialysis with HCV by Fibroscan and by biological scores (APRI, Forns and Fib-4), and to measure the correlation between these tests. Cross-sectional study including all chronic hemodialysis patients with hepatitis C virus, in two public hemodialysis centers of Fez. All patients were evaluated for liver fibrosis using noninvasive methods (FibroScan and laboratory tests). Subsequently, the correlation between different tests has been measured. 95 chronic hemodialysis were studied, twenty nine patients (30.5%) with chronic hepatitis C. The average age was 52.38 ± 16.8 years. Nine liver fibrosis cases have been concluded by forns score. Fibroscan has objectified significant fibrosis in 6 cases. On the other side APRI has objectified sgnifivant fibrosis only in 3 cases. The Fib-4 showed severe fibrosis in five cases. The results have been most consistent between APRI and Fib-4, followed by Fibroscan and Forns, then APRI and FibroScan.

## Introduction

Hepatitis C virus (HCV) is the most common viral infection in chronic hemodialysis patients, with a high prevalence and increased risk of developing cirrhosis [1, 2]. Liver biopsy is widely practiced and accepted. However, the chronic hemodialysis patients are a high risk of bleeding. Therefore, many alternative non invasive methods have been developed to assess liver fibrosis like Fibroscan and biological scores: fibrotest [3, 4], fibrometer [5], APRI, FIB-4 and Forns. Their interest has been demonstrated particulary in patients with hepatitis C infection and normal renal function. The aim of our study is to assess fibrosis liver in chronic hemodialysis patients with HCV infection by FibroScan and biological scores (APRI, Fib-4, and Forns), and to evaluate the correlation between these scores.

## Methods

This is a cross-sectional study, including all chronic hemodialysis patients with HCV infection followed in both public hemodialysis centers in Fez, between January and March 2011. Patients coinfected with HBV and HCV were excluded.

The 3rd generation ELISA was used to detect anti-HCV antibodies then the results were confirmed by PCR. FibroScan [6] and abdominal ultrasound was realized for all patients with HCV by a single examiner in functional explorations unit of the university hospital Hassan II in Fez. The endoscopy was realized in cirrhosis cases.

The diagnosis of liver cirrhosis was retained on clinical, biological and radiological data. The calculation is performed using the following formulas:

APRI = AST×100/ Platelets [7]

Forns =7.811-3.131 Log (Platelets) + 0.781 Log(GGT) + 3.467 Log (age)- 0.014(cholesterol) [8]

Fib-4 = (age x ASAT) / (Platelets x ALT)

Results are interpreted according to the threshold values for each score [9–12] (Table 1).

**Table 1:** Thresholds for each test in the diagnosis of significant fibrosis (6-9)

	Non significant fibrosis	Significant fibrosis
**Fibroscan**	≤7.1 kpa	>7.1 kpa
**APRI**	<0.4	≥0.95
**Forns**	<4.2	>6.9
**Fib-4**	<1.45	>3.25

The diagnosis of liver cirrhosis was accepted on FibroScan if the hepatic elasticity was higher than 12.5 kPa, and on the APRI if the score was higher than 1.

The measure of agreement between different non-invasive tests (FibroScan and biological scores) [13], was based on the study of KAPPA coefficient of Landis and Koch [14, 15]. This index is expressed as a numerical value corresponding to a degree of agreement between the parameters studied. The analysis of the degree of concordance across kappa index need to use the same coding for different biological and FibroScan scores based on a binary outcome "significant fibrosis" or "non-significant fibrosis" (Table 1).

## Results

Among 95 hemodilaysis, twenty nine patients (30.5%) had HCV. The average age was 52, 38 ± 16.8 years(23-81), with a female predominance( sex ratio 0.71). Hypertensive nephrosclerosis was the main cause of end-stage renal failure (37.9%), followed by glomerulonephritis and diabetic nephropathy in 20% and 8.8% of patients respectively. The etiology remained unknown in the third of cases. The average duration of hemodialysis was 84± 46 months (12-216). The FibroScan showed a significantly fibrosis in six cas (27.3%), all had higher liver elasticity (<12.5 Kpa). Twenty two patients had no significant fibrosis. According to APRI score, significant fibrosis (= F2) was noted in three cases, no significant fibrosis (>F2) in 18 cases and an non-interpretable score for the remaining patients.

Nine patients had a Forns< 6.9 (significant fibrosis) and five patients had Forns > 4.2 (insignificant fibrosis). The rest of patients had intermediate results. The Fib-4 test has eliminated hepatic fibrosis in 12 cases, only five patients had severe fibrosis (Fib-4 < 3.25) (Table 2). The diagnosis of liver cirrhosis was retained in eight patients. Doppler abdominal has showed signs of cirrhosis and/or portal hypertension in these patients. The esophageal varices were found only in two patients. In cirrhotic patients, the Fibroscan has confirmed the presence of severe fibrosis in five cases with sensitivity of 62.5%, and specificity of 95.2%. Among these patients, only Four had severe fibrosis by Fib-4 test, the rest were in the intermediate area. However, only two patients had hepatic cirrhosis at APRI score. Regarding the concordance between these scores and FibroScan, the results were most consistent between APRI and Fib-4, followed by Fibroscan and Forns, then Fibroscan and APRI. All these concordances were classified as moderate (Table 3). But the results were worse between APRI and Forns.

**Table 2:** Assessment of the liver fibrosis by non invasive methods (n = 29)

	Non significant Fibrosis	Intermediate area	Significant Fibrosis
**Fibroscan**	23	0	6
**APRI**	18	8	3
**Fib4**	12	12	5
**Forns**	5	15	9

**Table 3:** Correlation between biological scores and Fibroscan for fibrosis evaluation

Scores	kappa	Accord
**APRI-Forns**	0.088	Bad
**APRI-Fib 4**	0.492	Moderate
**Forns- Fib 4**	0.322	Moderate
**Fibroscan- APRI**	0.432	Moderate
**Fibroscan- Fib 4**	0.273	Moderate
**Fibroscan- Forns**	0.476	Moderate

## Discussion

According to recent guidelines, the assessment of liver fibrosis in patients with HCV infection without comorbidity is based on liver biopsy, FibroTest or FibroScan [16]. A recently published Meta-analysis [17] included the most important publications on the performance of FibroScan in the evaluation of liver fibrosis in chronic viral hepatitis C. There was a correlation between liver elasticity measured by Fibroscan and the degree of fibrosis on liver biopsy in all studies. Fibroscan was an effective test with an excellent specificity and sensitivity especially in detecting of significant fibrosis and cirrhosis. Other scores have been studied like fibrotest, fibrometre, hepascore, APRI, Fib-4 and Forns. The most studies have shown that APRI has an important negative predictive value to eliminate significant fibrosis and cirrhosis. As for Fib-4, the major advantage was to detect patients with severe fibrosis or cirrhosis. Forns was performant to eliminate minimum or insignificant fibrosis. Few studies have been conducted in chronic hemodialysis patients with HCV. The first study has evaluated the performance of APRI in 203 hemodialysis patients (HDC) infected with HCV [9]. Insignificant fibrosis was eliminated a threshold >0.4 with an 93% NPV. The threshold = 0.95 was in favor of significant fibrosis with an 66% PPV, and an area under the ROC curve of 0.801. Moreover, the threshold = 0.55 eliminates the presence of cirrhosis with an 99% NPV. In other study published in 2010, including 279 hemodialysis infected with HCV. Multivariate analysis showed that APRI was a predictor factor of significant fibrosis [10] with an area under the ROC curve at 0,83. Almost half of the patients undergoing liver biopsy were correctly diagnosed by adaptation of the threshold level of APRI, which joins the results of the previous study on the possibility of reducing the use of liver biopsy in this context. In a recent study published in 2011, including 284 hemodialysis with HCV [18]. They have showed that Fibroscan was better than APRI to detect significant fibrosis compared to liver biopsy. Indeed, the area under the ROC curve of Fibroscan was greater than that of APRI for predicting patients with significant hepatic fibrosis(= F2) (0.96 versus 0,84 p >0.001), patients with advanced liver fibrosis (= F3) (0.98 versus 0.93, p = 0.04), and patients with cirrhosis (F4) (0.99 against 0.92 p = 0.13), using the following thresholds 5.3, 8.3 and 9.2 respectively. In our study, liver biopsy was not realized, so we have compared the results obtained of various tests by measuring their respective concordance (kappa index).The most consistent results were those of the APRI and Fib-4, followed by those of Fibroscan Forns, then Fibroscan and APRI. These results were classified as moderate despite the small size of our patients. It's will be probably better by including an important workforce. In our series, eight cirrhosis cases have been confirmed. In these patients, Fibroscan was better than others scores in detecting severe fibrosis with a sensitivity at 62.5% and a specificity at 95.2%.These results are consistent with the literature data. The performance of Fibroscan would be better with a greater number of patients.

## Conclusion

In this study, 27% of patients infected with HCV had cirrhosis. This reflects the severity of the liver disease in this population. In hemodialysis patients, the noninvasive tests could be a suitable alternative to assess hepatitic fibrosis, given the higher risk of bleeding in liver biopsy. Despite the small sample of this work, the correlation between biological scores and Fibroscan was generally moderate. Fibroscan was the best test to detect liver cirrhosis.
